# Grey Matter Alterations Co-Localize with Functional Abnormalities in Developmental Dyslexia: An ALE Meta-Analysis

**DOI:** 10.1371/journal.pone.0043122

**Published:** 2012-08-20

**Authors:** Janosch Linkersdörfer, Jan Lonnemann, Sven Lindberg, Marcus Hasselhorn, Christian J. Fiebach

**Affiliations:** 1 Center for Individual Development and Adaptive Education of Children at Risk, Frankfurt, Germany; 2 German Institute for International Educational Research, Frankfurt, Germany; 3 Institute for Psychology, Goethe-University Frankfurt, Germany; 4 Donders Institute for Brain, Cognition and Behaviour, Radboud University Nijmegen, The Netherlands; Centre Hospitalier Universitaire Vaudois Lausanne - CHUV, UNIL, Switzerland

## Abstract

The neural correlates of developmental dyslexia have been investigated intensively over the last two decades and reliable evidence for a dysfunction of left-hemispheric reading systems in dyslexic readers has been found in functional neuroimaging studies. In addition, structural imaging studies using voxel-based morphometry (VBM) demonstrated grey matter reductions in dyslexics in several brain regions. To objectively assess the consistency of these findings, we performed activation likelihood estimation (ALE) meta-analysis on nine published VBM studies reporting 62 foci of grey matter reduction in dyslexic readers. We found six significant clusters of convergence in bilateral temporo-parietal and left occipito-temporal cortical regions and in the cerebellum bilaterally. To identify possible overlaps between structural and functional deviations in dyslexic readers, we conducted additional ALE meta-analyses of imaging studies reporting functional underactivations (125 foci from 24 studies) or overactivations (95 foci from 11 studies ) in dyslexics. Subsequent conjunction analyses revealed overlaps between the results of the VBM meta-analysis and the meta-analysis of functional underactivations in the fusiform and supramarginal gyri of the left hemisphere. An overlap between VBM results and the meta-analysis of functional overactivations was found in the left cerebellum. The results of our study provide evidence for consistent grey matter variations bilaterally in the dyslexic brain and substantial overlap of these structural variations with functional abnormalities in left hemispheric regions.

## Introduction

Developmental dyslexia is a severe difficulty in learning to read accurately and fluently that affects 5–17% of all children and often persists into adulthood (e.g., [1]). The most widely accepted explanation for the origin of this disorder is an underlying deficit in the representation and processing of speech sounds [2]–[4]. This phonological deficit is associated with deficient grapheme-phoneme decoding skills that are crucial in the beginning stages of reading development. Early difficulties in phonological reading in turn are assumed to exert a negative impact on the establishment of orthographic representations required for fluent and effortless reading. Although phonological deficits can be found in the majority of persons with reading difficulties, dyslexia is a heterogeneous condition [5], [6]. Several other sensorimotor deficits have been associated with the disorder resulting in the emergence of alternative theories describing phonological deficits as secondary consequences of basal auditory [7], visual [8], attentional [9] and/or motor problems [10].

Over the last two decades, numerous neuroimaging studies have examined functional brain abnormalities in persons with dyslexia of different ages and of different languages (for reviews see, e.g., [1], [11]). Converging evidence from these studies indicates that dyslexia is associated with functional underactivations in two posterior neural systems of the left hemisphere. The first system is located dorsally in a temporo-parietal region, including the posterior part of the superior temporal gyrus and the supramarginal and angular gyri of the inferior parietal lobule, and is assumed to be involved in grapheme-phoneme decoding. The second neural system is located ventrally in an occipito-temporal region including the extrastriate fusiform and the inferior temporal gyrus. This region, often referred to as the Visual Word Form Area (VWFA; [12]; but see also [13]), is considered to gradually specialize for the fast and effortless processing of familiar visual words or frequent letter strings within words during the first years of reading experience (e.g., [1]). A common developmental interpretation is that underactivations of the ventral system, representing inferior automaticity of the reading process, are the secondary result of primary dysfunctions in the dorsal system that are associated with problems in phonological reading [1], [14]. Two further areas have been proposed to exhibit differences between dyslexic and normal readers: Overactivations in bilateral inferior frontal gyri, along with other regions of the right hemisphere, have been postulated to represent compensatory processes (e.g., [11]). However, these results have been inconclusive and sometimes contradictory as other researchers reported no activation differences (e.g., [15]) or even underactivations (e.g., [16]) in the left inferior frontal gyrus in dyslexic readers. Finally, based on behavioral and brain activation differences between dyslexic and normal readers in tasks involving motor skills, the cerebellum has been proposed to play a major role in the origin of dyslexia (“cerebellar deficit hypothesis”; [10], [17]).

In three recent coordinate-based meta-analyses, the results of studies showing functional differences between dyslexic and normal readers have been quantitatively analyzed. To detect topographic convergence between studies, coordinate-based meta-analyses use the reported 3D coordinates of voxels of peak statistical difference as input foci. These foci are modeled as Gaussian probability distributions centered at the given coordinates and combined to three-dimensional brain maps that represent the likelihood of activation across studies, at each voxel, and that can be tested for significance. In the first meta-analysis, Maisog et al. [18] used activation likelihood estimation (ALE; [19]) to analyze nine studies comparing adult dyslexics with control participants in reading tasks involving words, pseudowords, or letters. In addition to functional underactivations in the occipito-temporal (i.e., ventral) and temporo-parietal (i.e., dorsal) reading systems described above, they also reported maxima of underactivations in the inferior frontal gyrus, precuneus, and thalamus of the left, and the fusiform, postcentral, and superior temporal gyri of the right hemisphere. Maxima of consistent overactivations were found in the thalamus and the anterior insula of the right hemisphere. In a second ALE meta-analysis that used foci from 17 original studies examining children and adults, Richlan and colleagues [20] reported similar results regarding underactivations of the two posterior reading systems. They also found that underactivations in the inferior frontal gyrus were accompanied by overactivations in the primary motor cortex and the anterior insula of the left hemisphere and bilateral subcortical structures. The authors interpreted these results as compensatory reliance on silent articulatory access to phonological word representations. In their recent meta-analysis using signed differential mapping (SDM; [21]), Richlan et al. [22] statistically compared the results of two meta-analyses including nine studies with adult and nine studies with child participants respectively. Meta-analyses of studies with both children and adults showed underactivations in the occipito-temporal reading system suggesting an early dysfunction of this region. For adult studies, this cluster of underactivation was relatively enlarged. With respect to the temporo-parietal system, underactivations in the superior temporal gyrus were only found for adult studies, while underactivations in the inferior parietal lobule were only found for studies of children.

In addition to differences in functional activation, several studies have examined neuroanatomical variations in dyslexic readers over the last years. First evidence came from a series of post-mortem studies on the brains of diagnosed cases of developmental dyslexia [23]–[26]. These studies demonstrated neuronal ectopias and architectonic dysplasias mainly in perisylvian regions of the left hemisphere which were interpreted as the results of disturbed neuronal migration during the prenatal stage. Further findings were an atypical symmetry of the planum temporale and deviations in thalamic structures consisting in disorganization and smaller neurons in the magnocellular layers of the lateral geniculate nuclei bilaterally and smaller neurons in the left medial geniculate nucleus. Galaburda’s case studies constitute an important step in the search for a neurophysiological basis of dyslexia. Nevertheless, the results have to be interpreted with caution as the number of analyzed brains was quite small (ranging from one to five between studies) and some of the individuals had further neurological issues.

In vivo neuroimaging studies using manual volumetric measurements that concentrated mainly on the perisylvian language regions of the left-hemisphere, especially the planum temporale, yielded inconclusive results (for reviews, see [27], [28]). The advent of newly developed analysis techniques such as voxel-based morphometry (VBM; [29]) enabled an objective localization of structural differences with high spatial resolution. Furthermore, VBM studies report results in standard stereotactic space, thereby allowing for directly comparing findings from different studies as well as linking them to the results of functional imaging studies. Over the last ten years, a growing number of VBM studies has been published and structural differences in grey matter density or volume between dyslexic and normal readers have been identified in several brain regions. Locations most frequently reported to exhibit grey matter reductions in dyslexic readers include bilateral posterior temporal, temporo-parietal, and occipito-temporal regions, and the cerebellum, thereby closely resembling the above mentioned reading systems that showed functional disturbances in dyslexics. These results have been narratively reviewed in two recent publications [27], [30] but up to date no quantitative meta-analysis objectively measuring convergence between the studies has been published. Furthermore, no attempt has yet been made to objectively compare the results of dyslexia studies using structural imaging to those using functional imaging.

The present study aims at closing this gap by quantitatively meta-analyzing the results of published VBM studies comparing dyslexic and control participants. A main goal was to objectively identify areas showing consistent neuroanatomical differences across the studies and to specify the broad anatomical description of grey matter abnormalities in dyslexic persons by providing results in a standard stereotaxic space. Quantitative meta-analyses of neuroimaging data allow to assess convergence between studies independently from differences regarding acquisition and analysis methods or heterogeneity of subject characteristics, which is especially important for the study of disorders as heterogenous as dyslexia. An additional goal of this study was to identify possible overlaps between structural and functional brain variations in dyslexia by quantitatively comparing the results of the meta-analysis of VBM studies to results of meta-analyses of functional imaging studies showing under- or overactivations in dyslexic readers. To this end, we conducted three ALE meta-analyses, i.e. one on VBM studies reporting grey matter reductions in dyslexic readers, including 62 foci from nine studies; a second meta-analysis of functional imaging studies reporting underactivations in dyslexics, including 125 foci from 24 studies, and a third meta-analysis of functional imaging studies reporting overactivations in dyslexics, including 95 foci from 11 studies.

## Materials and Methods

### Study Selection

For the VBM meta-analysis, relevant publications were identified by PubMed searches using the keywords dyslexia plus morphometry, voxel-based or voxelwise, as well as by exploration of additional publications from the reference lists of obtained articles. Studies were included in the analysis if they (1) used voxel-based morphometry, (2) reported group comparisons between dyslexic and control participants with respect to local changes in grey matter density or volume, (3) reported results in a standard reference space (Talairach or MNI) and (4) used the same threshold throughout the whole brain. Based on these criteria, nine studies were selected [16], [31]–[38]. Together, these studies included data from 277 participants (139 dyslexics and 138 controls) and reported 62 foci of grey matter reduction in dyslexic readers. Data are current with December, 2011. Only three of the nine studies reported grey matter increases in dyslexics. Five of the nine studies examined adult and four examined child or adolescent participants. Due to the small overall number of VBM studies, we included studies with participants from all age groups into the meta-analysis (cf. [20] for a similar approach). For an overview of the main characteristics of the included VBM studies, see Table 1.

**Table 1:** Overview of the studies included in the meta-analysis of VBM studies.

Year	First author	Native language	Dylexics		Controls		Modulated/unmodulated images	Threshold		Number of foci with	
			n	mean age	n	mean age		Voxel-level(height) p<	Cluster-level (extent) p< or no. of voxels/mm^3^	grey matter decrease	grey matter increase
2011	Raschle	English	10	5.9	10	5.5	modulated	0.001 unc.	0.01 corr.	5	0
2009	Pernet	French	38	27.2	39	27.8	modulated	0.005 unc.	20 voxels	15	5
2008	Kronbichler	German	13	15.8	15	15.4	modulated	0.005 unc.	100 mm^3^	11	8
2008	Steinbrinck	German	7	20.1	7	23.7	modulated	0.05 corr.	650 voxels	2	0
2007	Hoeft	English	19	14.4	19	14.4	modulated	0.01 corr.	0.01 corr	6	0
2005	Eckert	English	13	11.4	13	11.3	modulated	0.00001 unc.	0.001 corr.	5	0
2005	Vinckenbosch	French	13	17–30	10	–	unmodulated	0.01 corr.	0.05 corr.	1	1
2004	Brambati	Italian	10	31.6	11	27.4	modulated	0.05 corr.	25 voxels	9	0
2001	Brown	English	16	24	14	matched	unmodulated	0.05 unc.	0.05 corr.	8	0

For the selection of publications to be included in the meta-analyses of functional imaging studies, we aimed at consistency with the three previously published meta-analyses. The studies analyzed in Maisog et al.’s meta-analysis [18] represent a subset of the studies analyzed in Richlan et al.’s first meta-analysis [20] which in turn represent a subset of the studies analyzed in Richlan et al.’s second meta-analysis [22], with older studies [39]–[41] using the same participants being replaced by more recent ones [42]–[44]. Therefore, we included the 17 published papers [42]–[58] from Richlan et al.’s second [22] and four papers from Richlan et al.’s first meta-analysis [20], which were excluded in the second study because they examined adolescent participants [33], [59]–[60].

To identify further studies published in the meantime, we conducted a PubMed search with the same keywords (dyslexia plus imaging) as used by Richlan and colleagues and applied the same selection criteria as used by the previous meta-analyses [18], [20], [22]: Studies were included in the analysis if they (1) investigated reading or reading-related tasks in alphabetic languages (2) with visually presented words, pseudowords, or single letters in the participants native language, (3) reported group comparisons between dyslexic and control participants (4) in a standard stereotactic space (Talairach or MNI) and (5) used the same threshold throughout the whole brain. As we included studies with participants of all age groups in the VBM meta-analysis, we did not introduce any restrictions on the age of participants in the selection of functional imaging studies (in accordance with [20]; but in contrast to [18], [22]). Following these criteria, three additional studies could be identified [62]–[64].

According to the approach used by Richlan et al. [20], [22], if a study reported differences between dyslexic and control participants for more than one contrast (i.e., contrast of an activation condition against simple fixation or a low-level visual control task), only foci from one such contrast, typically involving the task putting highest demands on phonological processing, were included. For the 21 studies analyzed in the previously published meta-analyses, foci of under- and overactivation from the same contrasts used by Richlan and colleagues [20], [22] were selected, while the three newly selected studies reported foci of functional differences only for one contrast respectively. In total, the 24 selected studies included data from 736 participants (371 dyslexics and 365 controls) and reported 125 foci of underactivations in dyslexic readers. Out of these 24 studies, 11 reported 95 foci of overactivations. The main characteristics of the included functional imaging studies are listed in Table S1.

### ALE Meta-Analysis Procedure

We performed three separate ALE meta-analyses one on VBM studies reporting grey matter reductions in dyslexic readers, a second on imaging studies reporting functional underactivations in dyslexics, and a third on imaging studies reporting functional overactivations in dyslexics. As only three VBM studies reported in sum only 14 foci of grey matter increase in dyslexic participants, we did not perform an additional meta-analysis of those studies.

The meta-analyses were carried out using GingerALE software, version 2.1.1 ([65], available from http://brainmap.org/ale/). Prior to analysis, foci reported in Talairach space in the original studies were transformed into MNI space using the tal2icbm algorithm [66]. For each study, the reported foci were modeled as centers of three-dimensional Gaussian probability distributions with FWHMs determined on the basis of the number of participants in the respective study (see [67]). The probability values of all foci in a given study were then combined to a modeled activation (MA) map. To control for within-experiment effects, the MA value of each voxel was computed by taking the maximum probability associated with any one of the foci reported in the respective study [68]. Finally, voxel-wise ALE scores, representing the convergence between studies at the corresponding location, were calculated by taking the union of the individual MA maps.

In order to determine the probability of the ALE values under the null-hypothesis of spatial independence between the studies, a null-distribution was derived analytically (see [69]). Based on these probabilities, the ALE maps for the three meta-analyses were thresholded at a false discovery rate (FDR) of p<0.05 and a cluster threshold of 125 voxels. To examine possible overlaps between the significant clusters from the VBM meta-analysis and those from the functional meta-analyses, two formal conjunction analyses were performed by multiplying binarized versions of the thresholded ALE maps.

## Results

### ALE Meta-Analysis of VBM Studies

**Figure 1:**
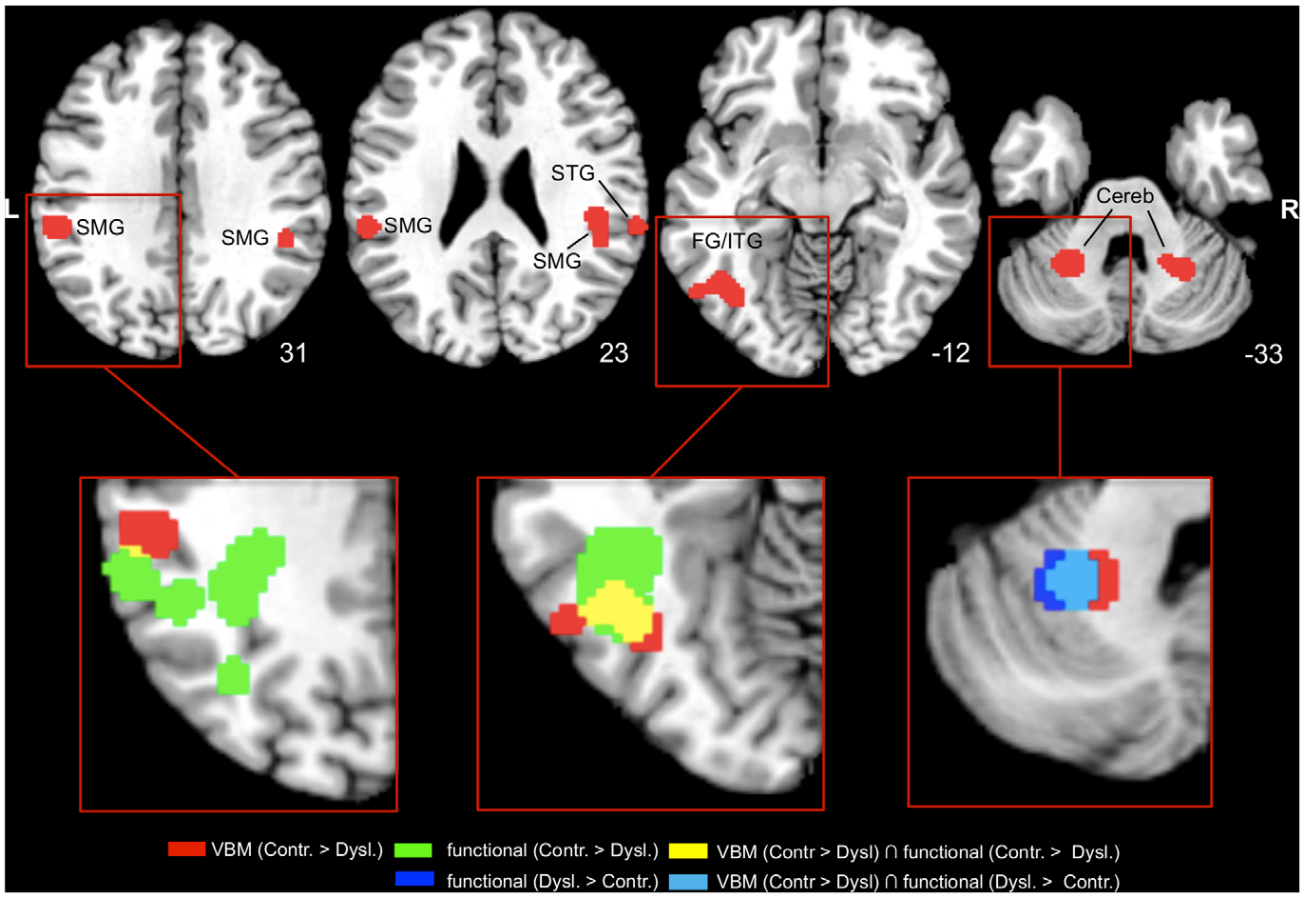
Results of the ALE meta-analysis of VBM studies and the conjunction analyses. Upper row–2D axial slices depicting the thresholded and binarized ALE map for the VBM meta-analysis (red) projected onto the Colin T1-template in MNI space. Images are presented in neurological convention (i.e., left = left) and MNI coordinates in the inferior-superior (Z) plane are provided with each slice. Lower row – cut-outs of the axial slices display overlaps (yellow) of the VBM meta-analysis (red) with the thresholded and binarized ALE map for the meta-analysis of functional underactivations (green) and overlaps (light blue) with the thresholded and binarized ALE map for the meta-analysis of functional overactivations (dark blue). SMG = supramarginal gyrus; STG = superior temporal gyrus; FG/ITG = fusiform gyrus/inferior temporal gyrus; Cereb = cerebellum.

In the meta-analysis of VBM studies reporting grey matter reductions in dyslexic readers, we found six clusters of significant convergence between the studies (see Table 2; Figure 1). The largest cluster was located in the fusiform gyrus of the left hemisphere extending into the left inferior temporal gyrus. Further clusters were found bilaterally in the supramarginal gyrus and in the cerebellum. In the right hemisphere, the supramarginal cluster extended into the parietal operculum and was accompanied by an additional cluster in the posterior portion of the superior temporal gyrus.

**Table 2:** Results of the ALE meta-analysis of VBM studies and the two conjunction analyses.

Region	MNI coordinates of local maxima			Cluster size (voxels)
	X	Y	Z	
*VBM meta-analysis (Contr. >Dysl.)*				
L supramarginal gyrus	−54	−34	30	156
R supramarginal gyrus	48	−40	26	143
	46	−30	24	a
R superior temporal gyrus	64	−34	18	131
L fusiform gyrus	−38	−66	−14	200
	−44	−60	−14	a
L inferior temporal gyrus	−56	−64	−10	a
L cerebellum	−26	−50	−32	158
R cerebellum	26	−54	−34	138
	18	−50	−38	a
*Conjunction analyses*				
VBM (Contr. >Dysl.)∩Imaging (Contr. >Dysl.)				
L supramarginal gyrus				1
L fusiform gyrus				95
VBM (Contr. >Dysl.)∩Imaging (Dysl. >Contr.)				
L cerebellum				99

a. subpeak within cluster.

### Overlap between Structural and Functional Alterations

Expectedly, the two meta-analyses of functional imaging studies reporting over- or underactivations in dyslexic readers yielded similar clusters of convergence as the previously published meta-analyses by Maisog et al. [18] and Richlan et al. [20], [22]. As the focus of the present study lies on the meta-analysis of VBM studies, these results will not be discussed in detail (see Table S2 for an overview).

The two formal conjunction analyses revealed three regional overlaps between the thresholded ALE maps from the VBM meta-analysis and those from the meta-analyses of functional imaging studies (see Table 2; Figure 1). The conjunction between the VBM meta-analysis and the meta-analysis of functional underactivations identified a large overlap of 95 voxels in the left fusiform gyrus (Figure 1, bottom row, middle panel). The supramarginal cluster from the VBM meta-analysis was located anterior and inferior, but in direct connection, to an extensive temporo-parietal cluster showing functional underactivation in dyslexics. There was a slight overlap of one voxel between the clusters in the left supramarginal gyrus (Figure 1, bottom row, left panel). The conjunction between the VBM meta-analysis and the meta-analysis of functional overactivations identified a large overlap of 99 voxels in the left cerebellum (Figure 1, bottom row, right panel).

## Discussion

The present study provides the first quantitative summary of published findings on grey matter variations in dyslexic readers, by conducting an ALE meta-analysis of nine published studies reporting grey matter reductions in dyslexics. The meta-analysis revealed six clusters of topographic convergence that were located bilaterally in temporo-parietal regions and in the cerebellum, as well as in occipito-temporal regions of the left hemisphere. Subsequent conjunction analyses identified overlaps with dyslexia-related functional underactivations in the fusiform and supramarginal gyri of the left hemisphere and an overlap with functional overactivations in the left cerebellum.

### Temporo-parietal Regions

The meta-analysis of VBM studies identified convergent grey matter reductions in temporo-parietal regions of both hemispheres. In the left hemisphere, one cluster with local maximum in the supramarginal gyrus was found. In the right hemisphere, a supramarginal cluster could also be identified that extended anteriorly into the parietal operculum. An additional cluster was found in the right superior temporal gyrus extending into the inferior parietal lobule.

The left parietal cluster corresponds well to the anatomical characterization of the dorsal posterior reading system which has been described by several authors [1], [70], [71]. This system is assumed to support the transformation of orthographic elements of visual words (graphemes) into associated phonological elements (phonemes). In the framework of the phonological theory of dyslexia, a dysfunction of this system has been proposed as the main biological basis of dyslexia [72]. Indeed, local grey matter volume in this region has been demonstrated to correlate with tasks involving phonological processing [35], [36] and these correlations could not be found for dyslexic readers [35]. Structural neuroimaging studies using manual methods in temporo-parietal regions concentrated mainly on symmetry measurements of the planum temporale and the planum parietale. While some studies reported greater symmetry or rightward asymmetry of the planum temporale and greater leftward asymmetry of the planum parietale, others could not confirm those findings (for reviews, see [27], [28]). Similar inconsistencies with regard to differences in asymmetry of temporo-parietal regions have been reported for other developmental disorders of speech and language, such as specific language impairment (SLI; [73], [74]) or stuttering [75]. Our VBM meta-analysis found grey matter reductions in dyslexic readers in areas corresponding to the planum temporale of the right and the planum parietale of both hemispheres. While an integration of results from micro- and macrostructural methods has to be interpreted with caution, our findings of grey matter decreases in the right planum temporale are not consistent with the proposal of greater rightward asymmetry in dyslexic readers. Likewise, as grey matter reductions affected the planum parietale not only of the left, but also the right hemisphere, the presently available VBM data also fail to support the proposed leftward asymmetry of the planum parietale.

On the basis of the present data, it remains unclear whether the grey matter reductions in temporo-parietal regions of dyslexic readers can be interpreted as the underlying neurobiological cause of deficient phonological processing skills in this population or should be better understood as the result of experience-dependent structural changes occurring in the course of school or preschool education. Raschle et al. [36] found grey matter alterations in temporo-parietal regions in familial dyslexic children before the onset of formal reading instruction. These findings suggest that the structural abnormalities are not the result but the cause of later reading problems. However, an alternative interpretation could be that abnormal phonological processing, possibly due to more basal auditory deficits (e.g., [7]), leads to secondary temporo-parietal grey matter alterations in dyslexic children even before reading instruction starts. The VBM meta-analysis also identified clusters of convergence in homologous areas of the right hemisphere, which conforms to the results of post-mortem studies by Galaburda and colleagues, showing that cortical abnormalities affected mainly perisylvian areas of the left hemisphere, but were always accompanied by corresponding abnormalities in the right hemisphere (see, e.g., [76]). Thus, our findings support the notion that bilateral anatomical variations represent a pre-existing neurobiological deficit of dyslexic readers, with left hemispheric variations underlying phonological processing problems. It can be hypothesized further that the parallel development of those bilateral regions might be under genetic control. Variations in genes involved in cortical development might result in microstructural cortical malformations through abnormal migration or maturation of neurons [77].

The left temporo-parietal cluster from the VBM meta-analysis was located directly anterior and inferior to an inferior parietal cluster from the meta-analysis of dyslexia-related functional underactivations, with an overlap of one voxel between the two clusters. The cluster of functional underactivations also included the posterior part of the supramarginal gyrus and extended into the angular gyrus and the superior parietal lobule. At its inferior border, this cluster of grey matter reductions was located in close proximity to a large occipito-temporal cluster of functional underactivations which extended into the superior temporal gyrus. In the right hemisphere, the superior temporal cluster from the VBM meta-analysis was located lateral and superior to a cluster from the meta-analysis of functional underactivations in the superior temporal gyrus.

To assess age-specific contributions to the relationship between structural and functional abnormalities, two additional meta-analyses were conducted, dividing the studies reporting functional underactivations in studies examining children and studies examining adults (for details, see Method S1; Table S3). While conjunction analyses of the results from the VBM meta-analysis with the results from both age-specific meta-analyses did not result in an overlap, the meta-analyses revealed that both child and adult studies contributed to the close connection between structural and functional deviations in left inferior parietal regions, with children’s underactivations being more wide-spread and extending into the angular gyrus and the superior parietal lobule. In contrast, the proximity to superior temporal underactivations in both hemispheres was driven by adult studies (see Table S4; Figure S1, left side). Taken together, the small overlap in the temporo-parietal system does not speak for a direct correspondence between the areas showing structural and functional abnormalities in dyslexic readers. Nonetheless, as the left-hemispheric cluster from the VBM meta-analysis was located in close connection to both the temporo-parietal and the occipito-temporal cluster showing functional underactivation in dyslexics, it is tempting to speculate that the anterior part of the temporo-parietal system might serve as a connectional hub and that structural deviations in this area might secondarily cause dysfunction of occipito-temporal and temporo-parietal regions and a disruption of functional connectivity between these areas that has been demonstrated in dyslexics [78]–[80].

### Occipito-temporal Regions

The largest cluster of dyslexia-related grey matter alteration identified by the VBM meta-analysis was located in occipito-temporal regions of the left hemisphere comprising mainly the fusiform gyrus and extending laterally into the inferior temporal gyrus. It thereby closely resembles the anatomical description of the ventral occipito-temporal reading system [70], [71] and includes the coordinates for the visual word form area (VWFA; x = 43, y = 54; z = 12; Talairach space) reported by Cohen et al. [12]. The occipito-temporal system is assumed to underlie the fast and effortless processing of printed text in experienced readers through direct mapping of orthographic word forms onto corresponding phonological representations (e.g., [81]). This specialization is assumed to develop gradually in the course of the process of learning to read, and functional underactivations of this region in dyslexic readers have been interpreted as the result of pre-existing functional and/or structural disruptions of the temporo-parietal reading system [1], [80].

As previously discussed, the results of our meta-analysis cannot answer the question whether grey matter microstructural abnormalities in dyslexics can be interpreted as the neurobiological cause of reading problems or should rather be understood as secondary, experience-dependent developmental changes. In contrast to the temporo-parietal system, where bilateral VBM effects were consistently observed, an occipito-temporal cluster of convergence between VBM studies could only be identified in the left hemisphere. If one assumes that disturbed neuronal migration should result in bilateral grey matter alterations in dyslexic readers, as suggested by the findings of post-mortem studies (cf. [76]), one might conclude that the observed structural abnormalities in left occipito-temporal regions represent the secondary result of disturbed reading and pre-reading experience. At least one VBM study, however, reported lower grey matter volume also in homologous areas of the right hemisphere [34]. Furthermore, [36] found lower grey matter volume in occipito-temporal regions of the left hemisphere in familial dyslexics in the last year before entering elementary school. As argued above, grey matter reductions might also have emerged in response to pre-school learning experiences, but it remains possible that occipito-temporal grey matter alterations may constitute a pre-existing neurobiological deficit, at least in a subgroup of dyslexics. Future, possibly longitudinal, studies examining structural abnormalities in persons suffering from or at risk for developmental dyslexia are needed to resolve this issue.

The conjunction analysis exhibited overlap between the occipito-temporal VBM cluster and an extensive occipito-temporal cluster showing reduced activation across functional imaging studies of dyslexic participants. The overlap comprised mainly the fusiform gyrus, including the above mentioned VWFA coordinates. Following the two explanatory approaches outlined above, microstructural alterations of grey matter in this region might either be the cause for a reduced functional responsiveness of the corresponding neural circuits or alternatively be the secondary result of abnormal input from and/or connectivity to temporo-parietal regions of the left hemisphere.

The age-specific meta-analyses of functional underactivations and conjunction analyses with the results from the VBM meta-analysis revealed that the occipito-temporal overlap between structural and functional deviations was driven by the studies examining adults, with an overlap of 82 voxels between the VBM meta-analysis and the meta-analysis of functional underactivations in adults. The meta-analysis of functional underactivations in children also resulted in a cluster in the fusiform gyrus, but it was located more anterior and inferior than the cluster of grey matter reduction (see Table S4; Figure S1, right side). The age-specific results highlight the increasing importance of structural and functional integrity of fusiform areas for the fast and effortless processing of words.

### Cerebellum

The VBM meta-analysis identified clusters of convergence in the cerebellum bilaterally. Traditionally, the cerebellum has been exclusively considered as a motor structure, controlling the coordination of movements. Recent research, however, points to an involvement of the cerebellum in higher cognitive functions by means of cerebro-cerebellar circuits targeting non-motor cortical regions (e.g., [82]). Indeed, cerebellar activation has been found by functional imaging studies employing a variety of tasks, including spatial processing, executive functions, working memory and language [83]. In addition to numerous PET and fMRI studies [84], the importance of the cerebellum for the processing of spoken and written language has been demonstrated by clinical studies showing phonological, semantic and syntactic impairments in patients with cerebellar lesions [85]. Accordingly, the cerebellum has been proposed to play an important role in the origin of dyslexia. The cerebellar deficit theory by Nicolson and colleagues [10] states that a mild dysfunction of the cerebellum leads to deficits in motor control and skill automatization in dyslexic readers. Supporting the theory, studies using different approaches have found structural abnormalities in the cerebellum to be a valid neurobiological marker for dyslexia [86]–[88]. A recent longitudinal VBM study demonstrated increased grey matter volume in the right anterior cerebellum in a group of dyslexic children following an eight-week reading intervention program [89]. On the other hand, meta-analyses of functional imaging studies comparing dyslexic and control participants did not reveal any reliable evidence for underactivations in the cerebellum [18], [20], [22]. Furthermore, motor problems seem to be present only in a subgroup of the dyslexic population (see, e.g., [6]) and it has been suggested that they only occur in dyslexic children with comorbid attention-deficit hyperactivity disorder (ADHD; [90], [91]). In their more recent work, Nicolson and colleagues [92] proposed that malfunctions in cortico-cerebellar loops might lead to procedural learning deficits which may affect both language and motor functions, either individually or in combination. An alternative explanation for morphometric alterations of cerebellar regions in dyslexic readers was raised by Ramus [93]. With reference to the work of Galaburda (for an overview see [76], [77]), he suggested that a core deficit in temporo-parietal regions of the cortex following disturbed neuronal migration may, under specific conditions, cause additional dysfunctions in subcortical regions, resulting in optional sensorimotor symptoms in a subgroup of dyslexic readers. Our finding of reliable structural alterations in homologous regions of the cerebellum highlights the importance of cerebellar deficits in dyslexia. The bilateral clusters of grey matter reductions were located mainly in the cerebellar lobule VI. Similar clusters were also found bilaterally by a meta-analysis of PET studies investigating single-word reading in normal adult participants [19] and in the right hemisphere by a meta-analysis of verbal working memory studies [94]. The clusters from the meta-analysis of single-word reading were located medial, posterior and superior, the cluster from the working memory meta-analysis superior to the clusters reported here. In a more recent study reporting several meta-analyses of functional imaging studies with different tasks, Stoodley and Schmahmann [83] also found bilateral clusters in the cerebellar lobule VI for verbal working memory and language tasks, with clusters being located more lateral and posterior. In the same study, also for motor tasks a right lateralized cluster (but note that all the tasks were finger-tapping tasks performed with the right index finger) in similar regions was reported, that extended from the anterior lobule V. Thus, the origin and implications of the grey matter reductions in the cerebellum found in the meta-analysis of VBM studies remain unclear. Further studies are needed to explore these questions in more detail.

In the left cerebellum, a large overlap between the VBM meta-analysis and the meta-analysis of functional overactivations in dyslexic readers was identified by the conjunction analysis. Additional meta-analyses examining age-specific contributions to the overlap could not be performed in this case, due to the limited number of studies reporting functional overactivations. The overlap between structural reduction and functional overactivation seems counterintuitive as one would rather assume that structural alterations of grey matter would result in decreases of functional responsiveness in corresponding regions. We hypothesize that increased activation in cerebellar and other sub-cortical regions in dyslexic readers while processing reading-related stimuli might represent increased effort, possibly due to disorganization of cortico-cerebellar loops and/or the use of compensatory strategies involving subvocal articulation or verbal working memory.

### Inferior Frontal Regions

Our meta-analysis did not find any reliable evidence for grey matter differences between dyslexic and normal readers in inferior frontal regions. The role of these regions in dyslexia has been discussed controversially –– bilateral inferior frontal gyri have been found to exhibit functional overactivations in dyslexics by some researchers, while others reported no activation differences or even functional underactivations, mainly in the left inferior frontal gyrus. Some evidence for deviations in inferior frontal regions has been found in structural imaging studies: Brown et al. [31] reported grey matter reductions in dyslexic readers in the left inferior frontal gyrus. In support of this finding, some studies found grey matter density or volume in this region to be positively correlated with behavioral performance in tasks involving phonological processing [35], [38], [87]. In addition, neuroimaging studies using manual morphometric measurements reported rightward asymmetry of the inferior frontal gyrus [95] and a smaller left [86] and right [32], [86] pars triangularis in dyslexic readers. In contrast, most published VBM studies did not identify any significant differences between dyslexic and normal readers in inferior frontal gyri, not even when applying small volume correction [34]. Thus, grey matter deviations in inferior frontal regions do not seem to play a major role in the neurobiological origin of dyslexia, but might, at least in some dyslexics, occur as a secondary consequence of deficient input from posterior reading systems or as a reflection of compensatory cognitive processes.

### Limitations

Besides methodological issues associated with coordinate-based meta-analyses in general [96] and inevitable drawbacks of meta-analyses of neuroimaging data, such as differences between the analyzed studies regarding data acquisition and processing, the present meta-analysis has the following limitations: The diagnostic criteria that were applied to select dyslexic participants varied greatly between the analyzed studies. While most studies used deviation from the norm of a standardized reading test as main criterion (i.e., ranging from 0.67 to 2 SD below average between studies), often combined with an intelligence test to ensure that intelligence was in the normal range, some studies used the stronger discrepancy criterion (i.e., discrepancy between the individual test scores in a reading and an intelligence test) and yet others selected participants based on evidence for a family history of dyslexia or a childhood diagnosis. One study [36] examined participants with familial history of dyslexia in the year before entering school, at a point in time where a reliable diagnosis for dyslexia cannot be made. Thus, the symptoms and possibly also the neuroanatomy of dyslexic participants included in the analyzed studies have to be regarded as heterogeneous. A further issue concerns the variability between the analyzed studies regarding the age of participants, with mean ages ranging from 5 to 30 in the VBM meta-analysis. To determine possible developmental changes of structural differences between dyslexic and control participants in the course of formal reading instruction, a comparison between separate meta-analyses of studies examining children and those examining adults would have been of interest, but was not possible due to the small number of studies that would be available for each age group in this analysis. In addition, separate analyses of studies using unmodulated (indicating differences in concentration) and modulated images (indicating differences in volume) might have provided further information about the nature of the observed structural differences between dyslexic and normal readers, but again could not be performed at the moment due to the limited number of available studies using each method. Finally, only studies from countries with alphabetic languages were included limiting the generalizability of the results of our study to specific cultures.

### Conclusion

By quantitatively integrating across individual studies, our meta analysis provides a strong empirical basis for understanding the neuroanatomical changes underlying developmental dyslexia. Clusters of topographic convergence were found exclusively in posterior brain regions, specifically in bilateral temporo-parietal and left occipito-temporal regions, and bilaterally in the cerebellum. These areas correspond well to the anatomical characterizations of neural systems that have been proposed to play a role in the origin of dyslexia by previous research. The analysis of conjunctions between the results of the VBM meta-analysis and meta-analyses of functional neuroimaging studies demonstrates that structural alterations of the reading systems in the left cerebral hemisphere co-occur with functional underactivations in these systems. Functional overactivations co-localize with grey matter reductions in the left cerebellum, possibly reflecting the compensatory use of articulatory strategies in dyslexic readers. In sum, our data suggest strong convergence between structural and functional alterations in the left hemisphere of the dyslexic brain.

## Supporting Information

Figure S1 **Results of the age specific conjunction analyses.** Upper row –– cut-outs of axial slices display the temporo-parietal and occipito-temporal overlaps (yellow) of the VBM meta-analysis (red) with the meta-analysis of functional underactivations (green) as depicted in Figure 1, bottom row. Lower row –– cut outs of axial slices display the results of the conjunction of the VBM meta-analysis (red) with the meta-analyses of functional underactivations in adults (light blue) and children (dark blue) for the same regions. Overlaps between the VBM meta-analysis and the adults meta-analysis are depicted in magenta, overlaps between the age-specific meta-analyses are depicted in cyan. Images are presented in neurological convention (i.e., left = left) and MNI coordinates in the inferior-superior (Z) plane are provided.
(TIF)

Table S1 **Overview of the studies included in the meta-analyses of functional imaging studies.**
(PDF)

Table S2 **Results of the ALE meta-analyses of functional imaging studies.**
(PDF)

Table S3 **Overview of the studies included in the age-specific meta-analyses of functional underactivation.**
(PDF)

Table S4 **Results of the age-specific ALE meta-analyses of functional underactivation and the age-specific conjunction analyses.**
(PDF)

Method S1 **Supplementary method.**
(PDF)
