# Supplementary figures and images for: Grey Matter Alterations Co-Localize with Functional Abnormalities in Developmental Dyslexia: An ALE Meta-Analysis
Source: PLoS One. 2012 Aug 20;7(8):e43122. doi: 10.1371/journal.pone.0043122 (PMC3423424; doi:10.1371/journal.pone.0043122)

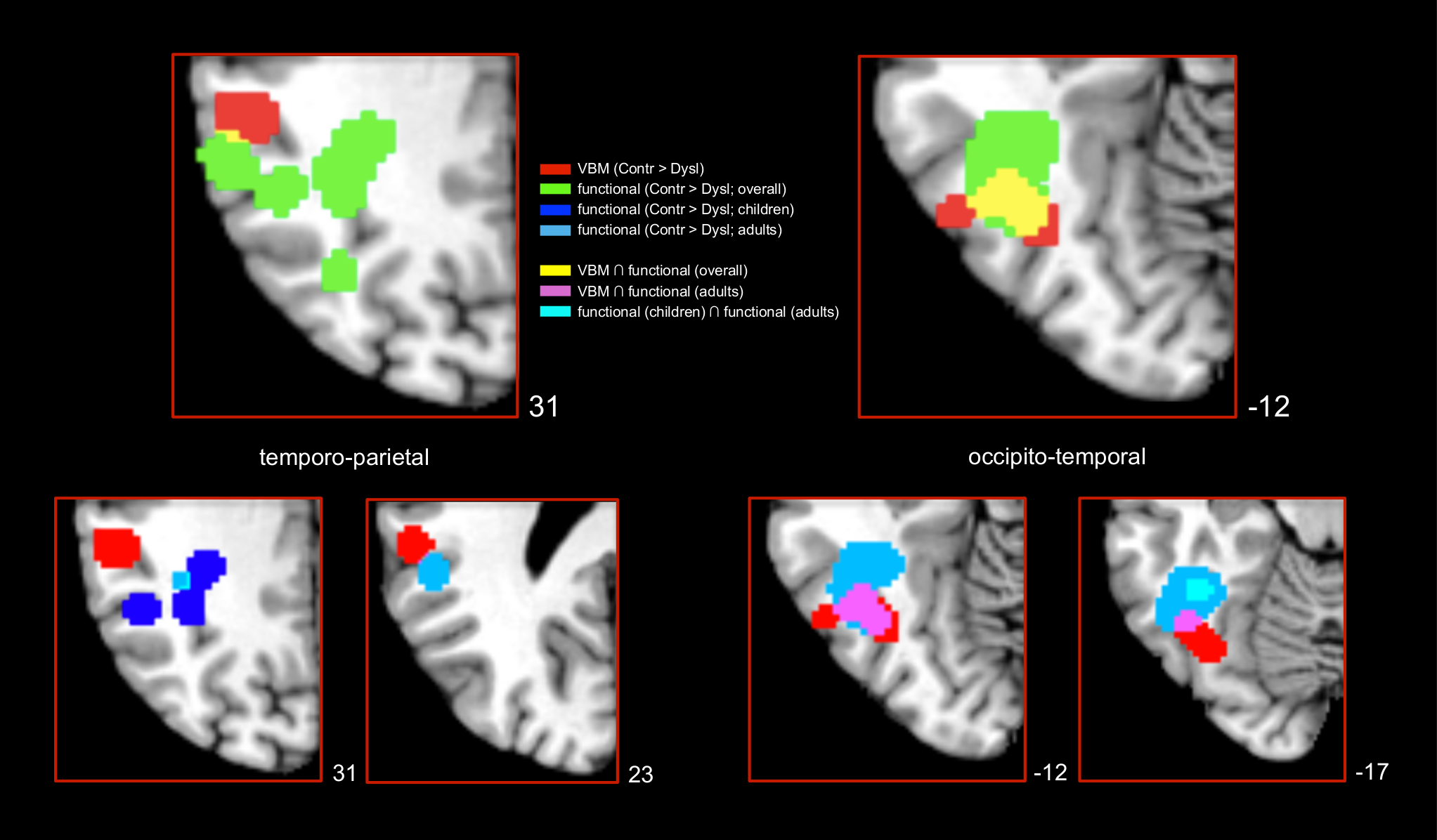

Supplement: Figure S1 — Results of the age specific conjunction analyses. Upper row –– cut-outs of axial slices display the temporo-parietal and occipito-temporal overlaps (yellow) of the VBM meta-analysis (red) with the meta-analysis of functional underactivations (green) as depicted in Figure 1, bottom row. Lower row –– cut outs of axial slices display the results of the conjunction of the VBM meta-analysis (red) with the meta-analyses of functional underactivations in adults (light blue) and children (dark blue) for the same regions. Overlaps between the VBM meta-analysis and the adults meta-analysis are depicted in magenta, overlaps between the age-specific meta-analyses are depicted in cyan. Images are presented in neurological convention (i.e., left = left) and MNI coordinates in the inferior-superior (Z) plane are provided. (TIF) [file pone.0043122.s001.tif]
