# Supplementary material for: Grey Matter Alterations Co-Localize with Functional Abnormalities in Developmental Dyslexia: An ALE Meta-Analysis
Source: PLoS One. 2012 Aug 20;7(8):e43122. doi: 10.1371/journal.pone.0043122 (PMC3423424; doi:10.1371/journal.pone.0043122)
Supplement: Table S2 — Results of the ALE meta-analyses of functional imaging studies. (PDF) [file pone.0043122.s003.pdf]

Supporting Information Table S2: Results of the ALE meta-analyses of functional imaging studies

Region	MNI coordinates of local maxima			Cluster size (voxels)
	X	Y	Z	
Contr. > Dysl.				
L supramarginal gyrus	-44	-44	40	1264
	-58	-48	38	a
	-50	-42	54	a
	-32	-40	34	a
	-60	-42	30	a
L superior parietal lobule	-34	-62	50	a
L middle temporal gyrus	-36	-68	34	a
L angular gyrus	-36	-60	40	a
L superior temporal gyrus	-52	-32	4	658
	-44	-26	8	a
R superior temporal gyrus	42	-22	6	276
	42	-32	6	a
	52	-34	4	a
L fusiform gyrus	-44	-50	-16	1453
L middle temporal gyrus	-60	-54	2	a
	-52	-50	12	a
	-46	-46	4	a
L superior temporal gyrus	-52	-42	22	a
L inferior frontal gyrus	-54	14	12	373
	-56	20	6	a
	-42	8	14	a
	-46	18	6	a
L middle frontal gyrus	-42	16	46	a
	-36	12	36	a
L precentral gyrus	-42	4	36	169
R cingulate gyrus	18	28	32	283
	10	30	26	a
Dysl. >Contr.				
L inferior frontal gyrus	-50	22	18	222
	-62	16	16	a
L superior frontal gyrus	-20	2	58	a
L middle frontal gyrus	-28	2	62	145
R middle frontal gyrus	24	38	22	301
	36	36	28	a
	32	44	34	a
L insula	-34	14	-2	126
	-34	20	-6	a
L postcentral gyrus	-50	-12	50	242
L precuneus	-24	-64	36	139
	-16	-66	40	a
L middle occipital gyrus	-18	-90	-8	129
L thalamus	-8	-18	16	295
	-8	-12	24	a
	-4	-12	6	a
R thalamus	16	-20	-6	307
L caudate nucleus	-20	0	20	265
L putamen	-20	-2	8	a
L insula	-28	-12	22	a
L putamen	-18	12	4	146
L caudate nucleus	-18	14	10	a
R caudate nucleus	16	14	6	301
	18	2	10	a
	18	12	18	a
L cerebellum	-28	-50	-32	143

a. subpeak within cluster.
