# Supplementary material for: Grey Matter Alterations Co-Localize with Functional Abnormalities in Developmental Dyslexia: An ALE Meta-Analysis
Source: PLoS One. 2012 Aug 20;7(8):e43122. doi: 10.1371/journal.pone.0043122 (PMC3423424; doi:10.1371/journal.pone.0043122)
Supplement: Table S3 — Overview of the studies included in the age-specific meta-analyses of functional underactivation. (PDF) [file pone.0043122.s004.pdf]

Supporting Information Table S3: Overview of the studies included in the age-specific meta-analyses of functional underactivation

Year	First author	Native language	Dylexics		Controls		Imaging	Task type	Contrast	Threshold		Number of foci with underactivations
			n	mean age	n	mean age				Voxel-level (height) p<	Cluster-level (extent) p< or no. of voxels / mm^3	
Children												
2011	Maurer	German	11	∼11.3	16	∼11.5	fMRI	Word/pseudoword/symbol string/picture identity judgment	words > symbol strings	0.01 unc.	0.05 corr.	3
2010	Blau	Dutch	18	9.3	16	9.4	fMRI	Letter-speech-sound integration	Interaction group x multisensory congruency	0.01 unc.	0.05 corr.	3
2009	Schulz	German	15	∼11.6	15	∼11.4	fMRI	Sentence evaluation	Sentence reading > fixation (age-matched)	0.001 unc.	5 voxels	9
2009	van der Mark	German	18	11.4	24	11.3	fMRI	Phonological lexical decision	Pseudohomophones > fixation	0.001 unc.	–	8
2008	Meyler	English	23	10.8	12	10.8	fMRI	Sentence evaluation	Sentence reading > fixation (pre-remediation)	0.002 unc.	10 voxels	6
2007	Booth	English	13	10.5	13	10.5	fMRI	Semantic association judgment	Related word pairs > fixation	0.001 unc.	15 voxels	1
2006	Cao	English	14	11.6	14	11.5	fMRI	Word rhyme judgment	Conflicting trials > fixation	0.001 unc.	15 voxels	6
2006	Hoeft	English	10	11.3	10	10.9	fMRI	Word rhyme judgment	Rhyme > fixation (age-matched)	0.001 unc.	10 voxels	6
2001	Temple	English	24	10.5	15	10.7	fMRI	Visual letter matching and line matching	Matching letters > matching lines	0.001 unc.	20 voxels	5
Adults												
2011	Pecini	Italian	13	24	13	22	fMRI	Rhyme generation	Rhyme generation > letter string viewing	0.05 corr.	$100mm^3$	4
2010	Richlan	German	15	16-20	18	16-19	fMRI	Phonological lexical decision	Pseudowords > fixation	0.005 unc.	20 voxels	15
2010	Wimmer	German	20	20.4	19	20.8	fMRI	Phonological lexical decision	Pseudowords > fixation	0.005 unc.	10 voxels	3
2009	Blau	Dutch	13	23.5	13	26.8	fMRI	Letter-speech-sound integration	Multisensory congruency	0.001 unc.	0.05 corr.	2
2006	Brambati	Italian	12	33	11	28	fMRI	Silent reading	Reading (words and peudowords) > false font string viewing	0.05 unc.	20 voxels	9
2005	McCrory	English	8	20	10	20.3	PET	Word reading and picture naming	Reading > false font string comparison	0.05 corr.	–	1
2002	Ingvar	Swedish	9	20-26	9	20-28	PET	Reading silently and aloud	Reading words silently > rest	0.001 unc.	–	3
2001	Paulesu	English, Italian, French	36	∼24	36	∼24	PET	Reading aloud	Reading (words and pseudowords) > rest	0.001 unc.	corr.	4
1997	Rumsey	English	17	27	14	25	PET	Reading aloud	Pseudowords > fixation	0.01 unc.	9 voxels	14
1996	Paulesu	English	5	25.2	5	27.2	PET	Letter pair rhyme judgment	Letter pair rhyming > shape similarity judgment	0.001 unc.	–	6
