# Supplementary material for: Grey Matter Alterations Co-Localize with Functional Abnormalities in Developmental Dyslexia: An ALE Meta-Analysis
Source: PLoS One. 2012 Aug 20;7(8):e43122. doi: 10.1371/journal.pone.0043122 (PMC3423424; doi:10.1371/journal.pone.0043122)
Supplement: Table S4 — Results of the age-specific ALE meta-analyses of functional underactivation and the age-specific conjunction analyses. (PDF) [file pone.0043122.s005.pdf]

Supporting Information Table S4: Results of the age-specific ALE meta-analyses of functional underactivation and the age-specific conjunction analyses

**Region**	**MNI coordinates of local maxima**			**Cluster size (voxels)**
	**X**	**Y**	**Z**	
Children				
L supramarginal gyrus	-50	-42	44	399
	-50	-38	54	a
	-58	-50	38	a
L superior parietal lobule	-40	-40	46	a
L angular gyrus	-48	-50	34	a
L superior temporal gyrus	-30	-40	32	128
	-34	-50	32	a
L middle temporal gyrus	-60	-54	4	236
	-60	-62	12	a
R supramarginal gyrus	56	-42	40	142
	48	-36	44	a
L fusiform gyrus	-42	-48	-24	161
L inferior frontal gyrus	-54	30	14	134
	-56	22	6	a
L middle frontal gyrus	-42	4	36	223
	-42	16	46	a
	-36	12	36	a
R inferior frontal gyrus	48	30	18	156
	36	36	18	a
Adults				
L supramarginal gyrus	-40	-44	38	246
	-28	-36	40	a
L superior temporal gyrus	-44	-26	6	444
	-50	-22	-2	a
L superior temporal gyrus	-50	-48	10	249
	-46	-46	4	a
	-52	-42	20	a
R superior temporal gyrus	42	-22	6	240
	42	-32	6	a
L fusiform gyrus	-44	-50	-16	583
L inferior temporal gyrus	-48	-62	-8	a
L inferior frontal gyrus	-56	12	12	252
	-46	18	6	a
L middle frontal gyrus	-28	-2	42	146
Conjunction analyses				
VBM (Contr. > Dysl.)∩Imaging (Contr. > Dysl. — children)				
–				–
VBM (Contr. > Dysl.)∩Imaging (Contr. > Dysl. — adults)				
L fusiform gyrus				82

a. subpeak within cluster.
