# Supplementary material for: Grey Matter Alterations Co-Localize with Functional Abnormalities in Developmental Dyslexia: An ALE Meta-Analysis
Source: PLoS One. 2012 Aug 20;7(8):e43122. doi: 10.1371/journal.pone.0043122 (PMC3423424; doi:10.1371/journal.pone.0043122)
Supplement: Method S1 — Supplementary method. (PDF) [file pone.0043122.s006.pdf]

Supplementary Method

To assess developmental aspects of the observed structure-function relationships, age-specific meta-analyses of the functional imaging studies were conducted. According to the approach used by Richlan et al. [1], the 24 studies reporting underactivations in dyslexic readers were divided into studies examining children (n=9), studies examining adults (n=10) and studies examining adolescents (n=5). Two additional meta-analyses for the studies examining children (47 foci; see Table S3 for an overview) and adults (61 foci; see Table S3 for an overview) were performed using the same ALE meta-analysis procedure as described in the main article. Studies with adolescents were excluded to allow for a more accurate distinction between child and adult studies. To examine possible overlaps between the significant clusters from the VBM meta-analysis and those from the age-specific meta-analyses, two additional conjunction analyses were performed by multiplying binarized versions of the thresholded ALE maps. A corresponding age-specific meta-analysis of functional overactivations could not be performed, because out of the 24 studies reporting underactivations in dyslexic readers only 11 reported overactivations, 4 of which were studies examining adolescents, thus leaving only 3 child and 4 adult studies.

References

1. Richlan F, Kronbichler M, Wimmer H (2011) Meta-analyzing brain dysfunctions in dyslexic children and adults. Neuroimage 56: 1735-42.
